# Bringing the value equation into play in value-based healthcare

**DOI:** 10.3389/fpubh.2026.1696854

**Published:** 2026-04-13

**Authors:** Borja García-Lorenzo, Arantzazu Arrospide, Iker Ustarroz-Aguirre, Ania Gorostiza, Itxaso Alayo, Teresa Acaiturri-Ayesta, Marisol Blanco, Izaskun Etxezarreta, Carolina Varela-Rodríguez, Elisa Gómez-Inhiesto, Ane Fullaondo, on behalf of the VOICE study group

**Affiliations:** 1 Biosistemak Institute for Health System Research, Bilbao, Basque Country, Spain; 2 Red de Investigacion en Cronicidad, Atencion Primaria y Promocion de la Salud RICAPPS-(RICORS), Instituto de Salud Carlos III (ISCIII), Madrid, Spain; 3 Department of Health, Basque Government, Vitoria-Gasteiz, Spain; 4 Osakidetza Basque Health Service, Ezkerraldea Enkarterri Cruces Integrated Health Organisation, Barakaldo, Spain; 5 Osakidetza Basque Health Service, Donostialdea Integrated Health Organisation, Donostia-San Sebastián, Spain; 6 Quality of Care Unit, Hospital Universitario 12 de Octubre, Madrid, Spain

**Keywords:** value-based healthcare, value equation, PACELY, efficiency, cost-effectiveness, QALY, HEOR, breast cancer

## Abstract

**Introduction:**

Value-based healthcare (VBHC) proposes a framework for managing healthcare systems, connecting health and economic outcomes to determine the value of healthcare. The value equation remains ambiguous, serving more as a theoretical framework than a practical decision-making tool. The key challenge lies in estimating and interpreting the value equation. The purpose of this study is to provide a methodological proof-of-concept to address this gap.

**Methods:**

A cohort of 330 patients diagnosed with breast cancer with a 12-month follow-up from two healthcare centres was used to illustrate the proposed approach. Patient-reported outcomes and economic-related outcomes (PROs and EROs) were collected. The numerator was defined as the patient-centred outcome-adjusted life years (PACELYs), a novel metric proposed here that combines PROs and survival, whilst the denominator was expressed in euros. Moving towards a marginal perspective, Incremental value (IV) and value curve were proposed as decision-making measures.

**Results:**

The mean PACELYs for healthcare centres A and B were between 69.85 and 73.24, and the costs for these centres were 12,129€–13,404€. The InIV showed that centre B generated an additional PACELY at 376€ compared to A, reflecting differences in organisational efficiency. The value curve showed variation in efficiency across VBHC thresholds, depending on the healthcare context.

**Discussion:**

This is the first proof-of-concept to estimate a value figure as a patient-centred efficiency measure for comparing healthcare providers within VBHC, with two pivotal transformations of the value equation: the use of PACELYs and the adoption of a marginal perspective, thereby positioning it as a decision-making tool in VBHC. The estimated figure will facilitate comprehensive benchmarking across centres and be applicable to other medical conditions. Further research should focus on designing value-based payment systems.

## 1 Introduction

Value-based healthcare (VBHC) aims to relate patient-centred outcomes (PCOs) with costs to estimate healthcare value. The VBHC framework evaluates healthcare delivery by assessing health outcomes generated per unit of healthcare spending (1, 2). As proposed by Porter, health outcomes have been delineated across diseases by the International Consortium for Health Outcomes Measurement (ICHOM) through the sets of patient-centred outcome (PCO) measures (3). This approach requires the strategic engagement of healthcare managers and technological resources to collect the PCOs, defined as disease-specific patient-reported outcomes (PROs) and clinical-related outcomes (CROs), and the economic-related outcomes (EROs), computed as healthcare resources used throughout the patient pathway and their corresponding unit costs. The well-known value equation for determining VBHC, introduced by Porter (4), defines VBHC as the ratio of PCOs to the cost of achieving them. However, previous literature (5–7) has argued that the value equation is still ambiguous and more of a theoretical framework than a useful decision-making tool. In particular, the VBHC has not been able to accurately measure the value of a given healthcare process due to the multidimensionality of the construct. To overcome this challenge, previous literature (8) has proposed qualitative methods for the estimation of the weights of the PCOs proposed by ICHOM (3). In addition, García-Lorenzo et al. (9) have proposed a quantitative methodological pathway for estimating the contribution of PCOs, thereby providing a single figure in the numerator of the value equation in the context of breast cancer. Whilst standard sets of PCOs are being developed across diseases, García-Lorenzo et al. (5) have highlighted the heterogeneity in resource-use counting and unit cost estimation methodologies as significant barriers to the implementation of fair and rigorous VBHC benchmarking across healthcare centres. Both previous studies (5, 9) have suggested, as further research, the need for joint estimation and interpretation of PCOs and EROs to estimate a single figure representing the value equation proposed by Porter. The aim of this study is to overcome the previous challenge by addressing a proof of concept for estimating the value equation, bringing established methods in the field of health economic evaluation to the emerging VBHC (10). Particularly, this study introduces and defines two pivotal transformations of the value equation: the use of patient-centred outcome-adjusted life years (PACELYs), as the combination of PCOs and survival, and the marginal perspective between two healthcare centres, importing methods from health economic evaluation (11, 12). These transformations are proposed to position it as a decision-making tool in VBHC.

## 2 Methods

### 2.1 Study design

This study was designed within the value-based healthcare for outcomes in breast and lung cancer in Europe (VOICE) community, a European healthcare cluster centre with a twofold objective: transitioning from theoretical foundations to practical implementation, and developing a VBHC delivery system. The study has been approved by the Ethics Committee of the Basque Country (protocol code PI2018107, approved on 5 December 2018).

The study design was conceived as a prospective multicentre cohort across six pilot sites. The study population consisted of patients diagnosed with early breast cancer between 2018 and 2020. A subset of data from the VOICE breast cancer cohort was used for this study. The cohort and recruitment are described in detail in García-Lorenzo et al. (5). This subset, recruited between January 2019 and June 2020, comprises data from the two Basque Country healthcare centres—*Ezkerraldea-Enkarterri-Cruces Integrated Health Organisation* and *Donostialdea Integrated Health Organisation*—both part of the *Osakidetza-Basque Health Service*. These centres were included because Osakidetza has implemented a patient-level costing information system, led by the *Ezkerraldea-Enkarterri-Cruces* organisation, which enables comparable cost-per-patient estimates based on care pathways. Tertiary hospitals use the same costing methodology to ensure the consistency of cost and outcome measurements required for VBHC benchmarking. Their selection was also driven by the availability of complete and harmonised data on PCOs and costs, essential for operationalising the value equation. Given the methodological aim of estimating no formal power calculation was required.

### 2.2 Data collection

Socio-demographic variables, PROs, and CROs were collected according to the International Consortium for Health Outcomes Measurements (ICHOM) standard set (3, 13): Socio-demographic variables were collected at baseline—defined as the biopsy date, whilst PRO and CRO information was collected at baseline and followed-up at 6 months.

As no standard set of economic outcomes was available for breast cancer, an *ad-hoc* set of resource uses was identified following the patient care pathway within the VOICE community. EROs, resource use, and unit costs were collected from the cost-per-patient information system for a 12-month follow-up. Resource use was grouped into specialist visits (e.g., gynaecology or oncology consultations), diagnostic procedures (e.g., mammography or biopsies), surgical interventions (e.g., conservative surgery or mastectomy), hospital stays (e.g., inpatient or ICU days), and treatments (e.g., chemotherapy or hormonotherapy sessions). Each category was linked to its corresponding unit costs, providing a structured overview of the economic components of care. The list of collected EROs can be found in TS1 of the Supplementary material.

### 2.3 Descriptive analysis

A descriptive analysis of PROs, CROs, and EROs of the pooled sample and across sites was performed, followed by statistical tests to assess differences across sites.

### 2.4 Value

Porter (4) defines the value of healthcare to patients provided by a healthcare centre for a particular disease as the ratio of the PCOs divided by the cost incurred to achieve those outcomes, as follows in Equation (1):


$$\text{Value}=\frac{\text{Patient}-\text{Centred}\;Ou\text{tcomes}\;(\mathrm{PCO})}{\text{Costs}}$$ (1)

Then, the numerator has been estimated using the composite indicator of patient-centred outcomes (CI-PCOs), according to García-Lorenzo et al. (9) as follows in Equation (2):


$$\mathrm{CI}-\mathrm{PC}O_{\mathrm{ijt}}=\sum_{k=1}^{k=K}w_{k}\cdot \mathrm{PC}O_{\text{kijt}}$$ (2)

where $\mathrm{CI}-\mathrm{PC}O_{\mathrm{ijt}}$ of patient *i* admitted at healthcare centre *j* at time *t* is the weighted sum of the ${\mathrm{PCO}}_{\text{kijt}}$ , whilst $w_{k}\;$ represented the weight of the *k*^th^ element of the $\mathrm{PC}O_{\mathrm{ijt}}$ . The $\mathrm{CI}-\mathrm{PC}O_{\mathrm{ijt}}$ ranges from 0 to 100. The PCOs included in the CI-PCOs correspond to those defined in the ICHOM breast cancer standard set (3, 13), covering functioning domains (physical, emotional, cognitive, and social functioning), symptom domains (pain, fatigue, insomnia, breast, and arm symptoms), and work-related functioning. These outcomes were measured using validated instruments collected longitudinally in routine clinical care (EORTC QLQ-C30, BR23, LMC21, BREAST-Q, and FACT-6). The associated weights $w_{k}\;$ were obtained by estimating multivariable regression models in which standardised PCOs were regressed on health-related quality of life (HRQoL) as a proxy outcome; statistically significant standardised coefficients were then normalised to derive the final weights. The PCOs that contributed significantly to the CI-PCOs were pain, physical functioning, emotional functioning, ability to work, and either breast or arm symptoms, depending on the time structure. Further methodological details are available in García-Lorenzo et al. (9).

Analogous to health economic evaluation and following the widely used conceptual definition of quality adjusted-life year (QALY), (14) which combines HRQoL with follow-up interval and survival, this paper proposes introducing and estimating a novel metric, patient-centred outcome-adjusted life years (PACELYs), as a combination of PCOs with follow-up interval and survival. Therefore, PACELYs of a patient *i* admitted in the healthcare centre *j* during the period $\left[t_{0},t_{1}\right]$ is calculated as follows in Equation (3):


$$\text{PACEL}Y_{\mathrm{ij}[t_{0},t_{1}]\;\;}=\frac{\left(\mathrm{CI}-\mathrm{PC}O_{\mathrm{ij}t_{0}}+\mathrm{CI}-\mathrm{PC}O_{\mathrm{ij}t_{1}}\right)}{2}(t_{1}-t_{0})$$ (3)

where $\mathrm{CI}-\mathrm{PC}O_{\mathrm{ij}t_{0}}$ and $\mathrm{CI}-{\mathrm{PCO}}_{{\mathrm{ijt}}_{1}}$ denote the estimates of CI-PCOs for patient *i* admitted at healthcare centre *j* evaluated at baseline (*t*₀) and follow-up (*t*₁), respectively. This transformation allows the value equation to incorporate both patient-centred outcomes and survival, moving beyond the static interpretation of CI-PCOs alone. Thus, the value of healthcare $V_{j}$ of the healthcare centre *j* is estimated as follows in Equation (4):


$$V_{j}=\frac{{\bar{\text{PACELY}}}_{j\;\;}\;\;\;}{{\bar{C}}_{j\;\;}}$$ (4)

where the numerator, ${\bar{\text{PACELY}}}_{j}$ , was defined as the mean PACELYs of patients admitted to the healthcare centre *j*, and the healthcare costs ${\bar{C}}_{j}$ , in the denominator, were defined as the mean costs generated by patients in healthcare centre *j* over the patient pathway, being calculated using a bottom-up (15) approach based on the cost-per-patient information system. This equation preserves the original structure of Porter’s value formulation. The contribution of this study lies in operationalising this ratio by constructing PACELYs that integrate patient-centred outcomes with survival and follow-up time. In this framework, PACELYs serve as the unit of health outcomes—analogous to QALYs in conventional economic evaluations—thereby enabling the value equation to function as a measurable and interpretable efficiency metric.

The value plane represents the *V_j_*-computed as the mean PACELYs for healthcare centre *j* divided by the mean cost of healthcare for centre *j*, was represented on the value plane. According to the value plane, the curve showing the lowest slope will identify the healthcare centre providing the highest value *V_j_*.

### 2.5 Incremental value

In health economic evaluation, the incremental approach has been widely proposed to inform appropriate decisions about either the coverage of certain interventions by insurers, or the design of payment schemes to pay for them (11). In analogy to this framework (11, 12), this study proposes to incorporate the incremental approach into the value equation when comparing healthcare services. This incremental perspective has not previously been applied within VBHC and enables the value equation to be used for comparative efficiency assessment, analogous to the role of incremental cost-effectiveness ratio (ICER) in health economic evaluation. To consider the added value between centres, the value equation previously defined as $V_{j}$ might be transformed into an incremental measure proposed as the incremental value (IV), between two healthcare centres, HC_A_ and HC_B_, taking HC_A_ as the reference, defined as follows in Equation (5):


$$IV_{B,A}=\frac{{\bar{C}}_{B}-{\bar{C}}_{A}}{{\bar{\text{PACELY}}}_{B}-{\bar{\text{PACELY}}}_{A}}$$ (5)

where $IV_{B,A}$ is the incremental value between HC_A_ and HC_B_. $\;{\bar{C}}_{A}$ and ${\bar{C}}_{B}$ are the mean costs of patients *i* admitted in both healthcare centres, respectively; whilst ${\bar{\text{PACELY}}}_{A}$ and ${\bar{\text{PACELY}}}_{B}$ are defined as the mean PACELYs of patients *i* admitted in the healthcare centre *A* and *B*, respectively. It is important to note that this reformulation of the value equation does not involve any mathematical inversion. The modification simply consists of exchanging the position of the numerator and the denominator to express costs relative to PACELYs. Then, *IV* can be interpreted in the same way as an ICER: as the additional cost required to obtain one extra PACELY when comparing two centres. The incremental value plane represents the incremental costs and the incremental PACELYs on the *Y*-axis and *X*-axis, respectively, where the $IV_{B,A}$ can be represented. In this example, the healthcare centre A is considered as the reference centre, whilst Supplementary Figure S1 provides a theoretical illustration of the incremental value plane. In the event that the $IV_{B,A}$ is located in the southeast (or northwest) quadrant, the interpretation process is straightforward. The $IV_{B,A}$ indicates that healthcare centre B is providing a greater number of PACELYs at a lower cost than A. This suggests that healthcare centre B dominates A in terms of value. In the northeast (or southwest) quadrant, the $IV_{B,A}$ indicates that the healthcare centre B will provide more PACELYs at a higher cost than A. In this scenario, the $IV_{B,A}$ must be evaluated in relation to a defined ceiling value, which determines whether the value added by healthcare centre B (A) represents efficient utilisation of limited resources related to A (B). This ceiling value might be defined as the VBHC threshold and it should represent the opportunity cost of generating an additional PACELY.

### 2.6 Sensitivity analysis

To represent the uncertainty of V estimation in the incremental value plane, non-parametric bootstrap methods were applied using different samples, assuming that initial samples from each centre represented the real population. A total of 1,000 simulations were carried out, where new samples of patients were created by selecting patients (with replacement). The bootstrapping method allowed estimating the 95% confidence ellipses for V using the variance–covariance matrix estimated in the joint analysis of costs and PACELYs carried out using seemingly unrelated regression (16) adjusted for baseline patient characteristics that may significantly differ between centres. Similar to the cost-effectiveness acceptability curve used in health economic evaluation, a value curve (12) was drawn to summarise the impact of uncertainty on V. This technique has been proposed as a means of addressing decision-making issues pertaining to confidence intervals from a marginal perspective (17). Subsequently, the value curve was expressed as the probability that healthcare centre B would be considered more efficient than A, in terms of value, depending on potential values of the VBHC threshold.

All analyses were performed using R version 4.2.2 and STATA 18. Statistical significance was set at a *p*-value of < 0.05.

## 3 Results

### 3.1 Descriptive analysis

A total of 330 patients accepted to participate, whilst 298 (90%) completed the questionnaires. The average age was 58 years (SD = 12); 37.2% reported comorbidities, and 65.3% had postmenopausal status. Baseline patient characteristics were comparable between centres. There were no significant differences in age at diagnosis (*p* = 0.166), educational level (*p* = 0.148), menopausal status (*p* = 0.483), or comorbidity (*p* = 0.103), indicating that the two populations were similar in terms of case mix. The sample description is shown in Supplementary Table S2. Supplementary Table S2 also includes the statistical description of the PROs, CROs, and EROs.

### 3.2 Value

**Figure 1:**
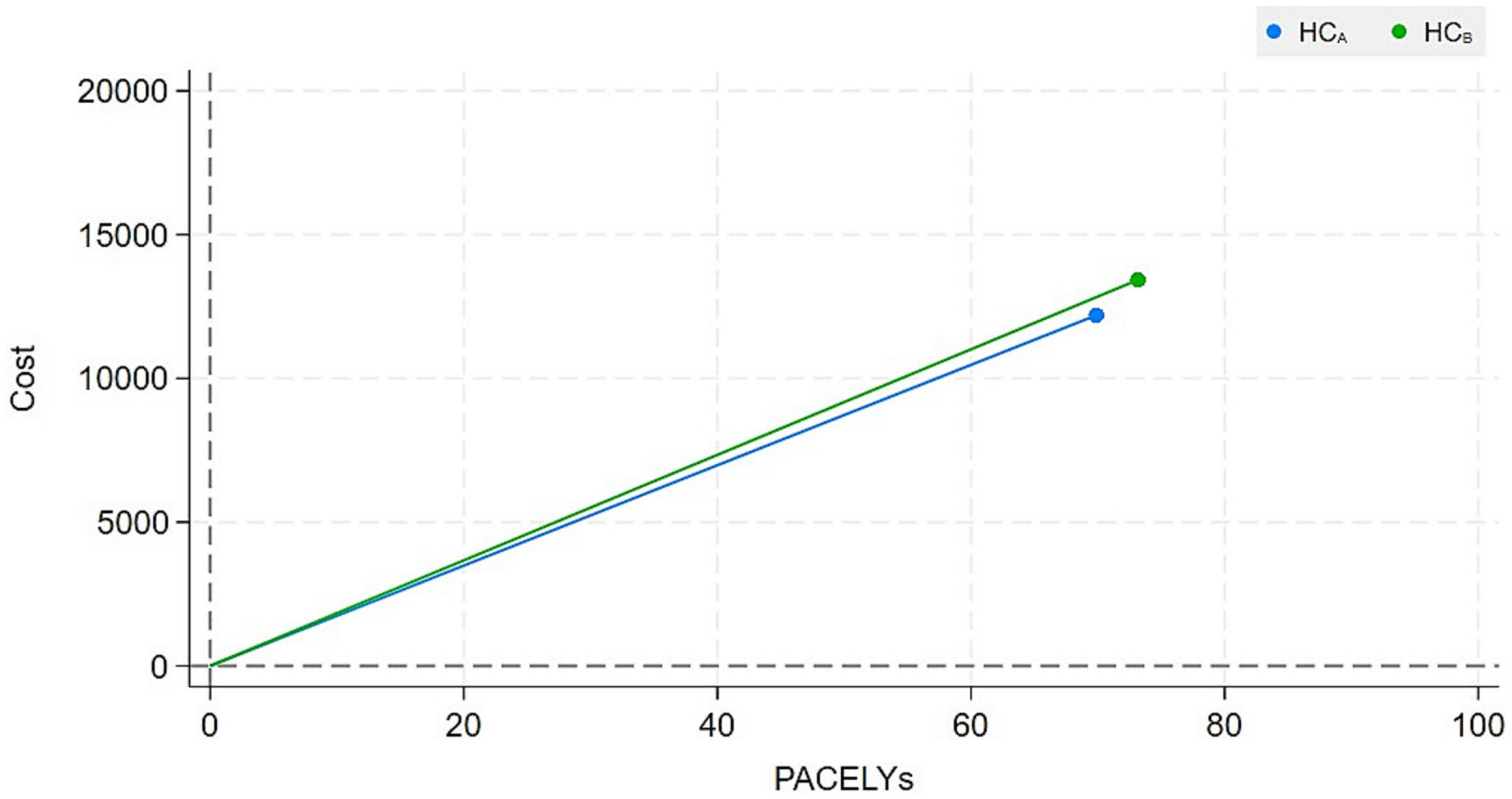
Value plane. PACELYs, patient-centred outcome-adjusted life years; HC_A_, healthcare centre A; HC_B_, healthcare centre B.

The mean PACELYs and costs of healthcare centres A and B for the provision of breast cancer healthcare were estimated at 69.85 and 73.24 PACELYs, and 12,129 and 13,404 euros, respectively. Subsequently, healthcare centre B provided a greater number of PACELYs at a higher cost than A. *V_A_* and *V_B_* were estimated at 0.0058 and 0.0055, respectively, indicating that healthcare centre A provided a slightly higher value than healthcare centre B. The slope of the curve linking the zero point to *V*_A_ is lower than that of *V*_B_. The results are shown in detail in Table 1, whilst Figure 1 illustrates the value plane where *V_A_* and *V_B_* are represented.

**Table 1:** Value-based healthcare results.

Healthcare centres	*N*	Mean (sd) PACELYs	Mean (sd) costs	*V*	IV
HC_A_	51	69.85 (7.54)	12,129 (6,600)	0.0058	376
HC_B_	240	73.24 (6.11)	13,404 (9,726)	0.0055	
Incremental (HC_B_ – HC_A_)	n.a.	3.39	1,275	0.0003	

PACELYs, patient-centred outcome-adjusted life years; *N*, sample size; sd, standard deviation; *V*, value; IV, incremental value; n.a., non-applicable; HC_A_, healthcare centre A; HC_B_, healthcare centre B.

### 3.3 Incremental value

**Figure 2:**
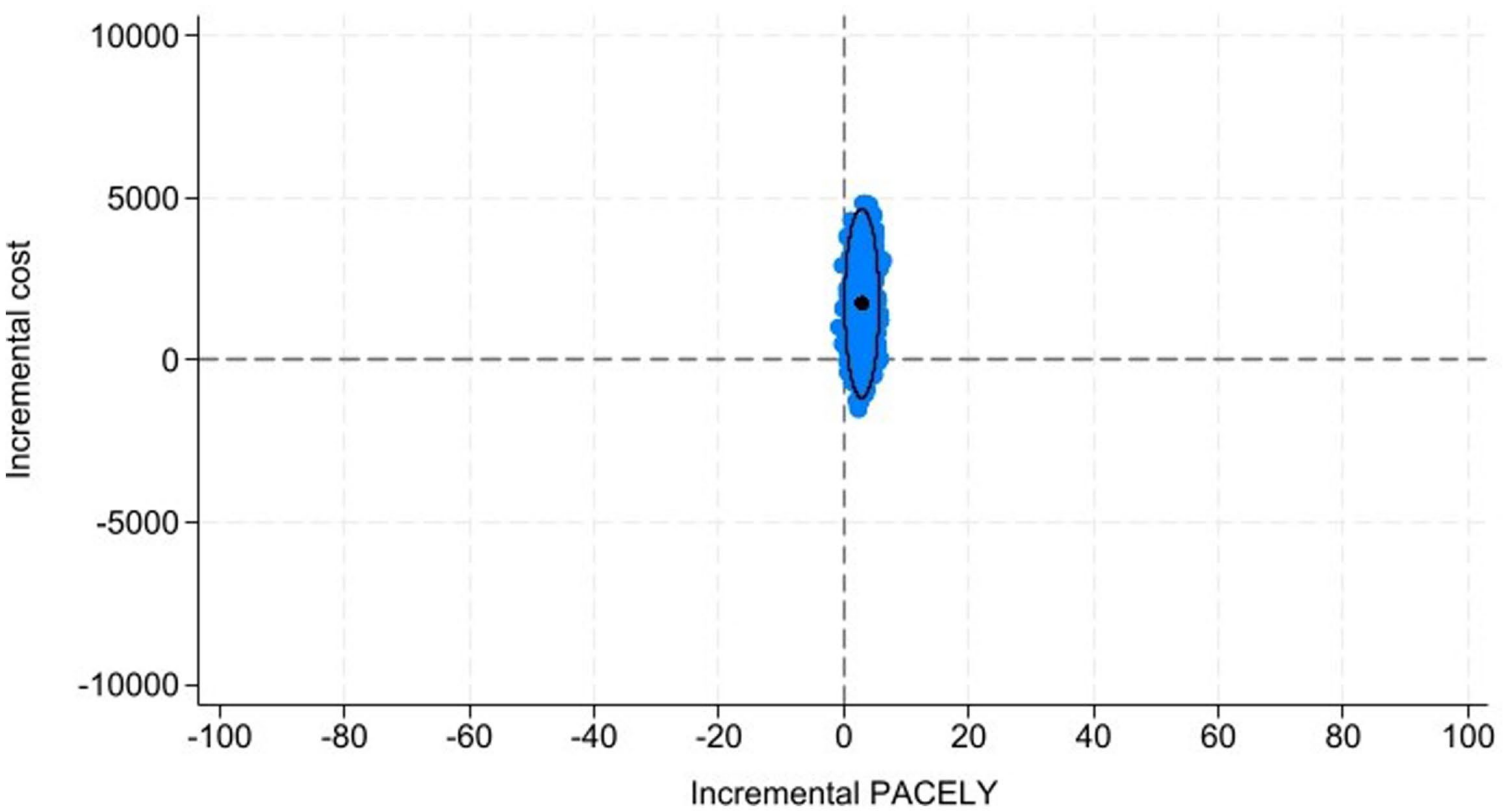
Incremental value plane. PACELYs, patient-centred outcome-adjusted life years, IV = 376 Euros per PACELYs, IV simulations, 95% confidence ellipse.

The $IV_{B,A}$ was estimated at 376 euros, indicating that centre B was delivering more PACELYs but at a higher cost than centre A. From a marginal perspective, this means that centre B incurs an incremental cost of €376 for each additional PACELY gained relative to centre A. In practical terms, the IV expresses how many additional euros centre B needs, compared with centre A, to produce one extra PACELY. Unlike an ICER—which compares alternative technologies—the IV evaluates the efficiency with which different centres transform resources into patient-centred outcomes, providing a measure of organisational performance rather than the cost-effectiveness of interventions. Table 1 shows a detailed overview of the results, whilst Figure 2 illustrates the incremental value plane, which depicts $IV_{B,A}$ and its corresponding 95% confidence ellipses are represented.

### 3.4 Sensitivity analysis

**Figure 3:**
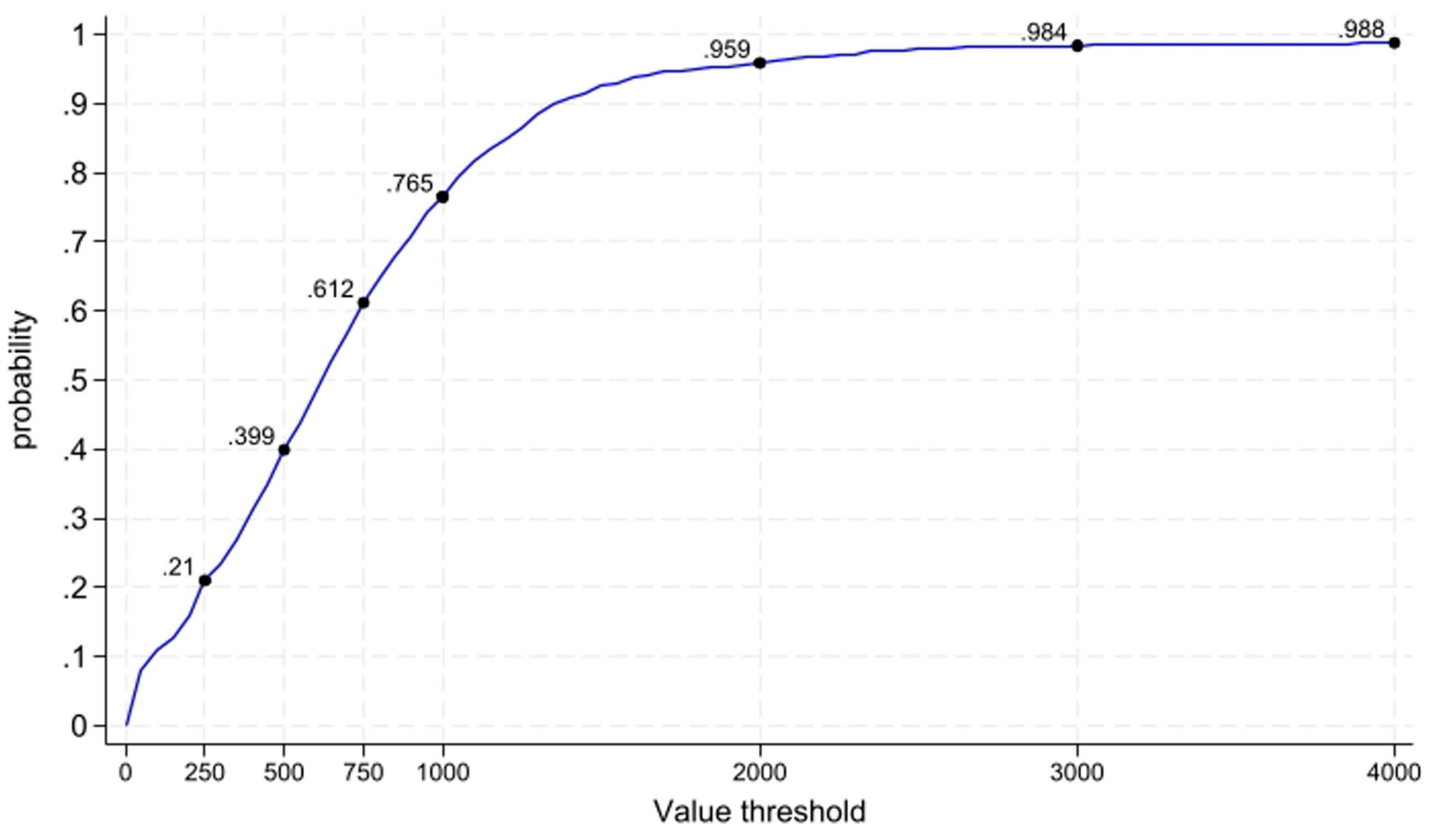
Value curve.

Figure 3 illustrates that the majority of the IV simulations into the 95% confidence ellipse fell within the northeast quadrant, showing that the healthcare centre B was providing a greater number of PACELYs at a higher cost compared to A, with a probability of 86.9%. The remaining IV simulations were located in the southeast quadrant, showing that healthcare centre B provides more PACELYs at a lower cost than healthcare centre A. This suggests that healthcare centre B dominates A in terms of value with a probability of 13%. More generally, Figure 3 illustrates the value curve, which shows that, across a range of theoretical VBHC thresholds, the probability of healthcare centre B offering better value in terms of efficiency compared to A varies. The value curve summarises how this probability changes across hypothetical VBHC thresholds.

## 4 Discussion

One of the main challenges with VBHC is combining health and economic outcomes using the value equation to reach a single value figure. Porter (4) introduced VBHC by defining healthcare value as the ratio of PCOs to the costs incurred in achieving those outcomes. However, García-Lorenzo et al. (5) have previously claimed that the definition of the value equation is still ambiguous, more of a theoretical framework than a practical decision-making tool. This study addresses the aforementioned challenge and that of jointly interpreting PCOs and costs in VBHC. Specifically, it aims to estimate and interpret the value equation suggested by Porter to bring it into play as a decision-making tool in VBHC.

The numerator was estimated following the proposed method in the literature (9) and defined as a multidimensional measure underpinned by a composite indicator, whilst the denominator was addressed following a bottom-up (15) approach and exploiting the cost-per-patient information system to calculate the cost per patient.

The novelty of this research does not lie in redefining Porter’s value equation—which indeed remains a ratio of outcomes to costs—but in its operationalisation through two pivotal transformations. First, we estimate PACELYs, which extend the CI-PCOs by incorporating survival and follow-up time, thereby creating a dynamic patient-centred outcome measure analogous to the QALY but grounded in disease-specific PROs. Second, instead of relying solely on the simple ratio, we introduce the IV and the value curve to enable a marginal, comparative efficiency assessment between healthcare providers. These tools incorporate uncertainty and allow the evaluation of value across hypothetical VBHC thresholds, providing the decision-making evidence required for value-based payment frameworks. The value figure was computed as the mean PACELYs divided by the mean cost. Furthermore, a value plane, an incremental value plane, and a value curve were finally suggested to illustrate the proposed measures.

The mean PACELYs and costs for healthcare centres A and B were estimated at 69.85 and 73.24 PACELYs, and 12,129 and 13,404 euros, respectively. Then, healthcare centre B provided more PACELYs at a higher cost than A. V_A_ and V_B_ were estimated at 0.0058 and 0.0055, respectively, indicating that healthcare centre A provides a slightly higher value than B. It is interesting to note that the differences in PACELYs and costs between the healthcare centres might be affected by the distribution of the patients’ severity and differences in unit costs. The $IV_{B,A}$ was estimated at 376 euros, indicating that healthcare centre B generated an additional PACELY at 376 euros compared to A. Interpreting the IV as the additional cost required to obtain one extra PACELY provides a clear and intuitive measure of relative efficiency between the centres.

As in health economic evaluation, the interpretation of a marginal value measure depends on the existence of a ceiling value that reflects the opportunity cost of generating an additional unit of the outcome. Although a body of empirical research has estimated CE thresholds worldwide in the context of health economic evaluation (18), there is currently no empirical basis for defining an equivalent VBHC threshold. This conceptual limitation does not restrict the interpretability of the IV itself, which—analogous to the ICER—remains informative even in the absence of a threshold. The value curve explicitly shows how the probability of one centre offering better value than another varies across hypothetical thresholds, thereby avoiding reliance on a single decision rule.

Beyond this conceptual issue, the study also has additional limitations. The study followed up on both PROs and EROs at 6 and 12 months, respectively. The temporal lag between PROs and EROs may introduce bias into the value estimation process. Then, it would be optimal to obtain PROs at 12-month follow-up. In this study, the PROs collected at the 6-month interval were utilised as a proxy for the 12-month PROs; however, as the aim of this paper was to provide a proof of concept of the estimation of the value figure using the value equation, the potential changes in the results would not affect the application of the proposed method. Another limitation was related to the discrepancy in sample sizes between the two healthcare centres, which may affect the estimation of uncertainty in mean values when using bootstrap techniques. Therefore, it is recommended that similar sample sizes be used to ensure a more precise estimation of uncertainty. The empirical application using breast cancer patients is illustrated rather than representative of the broader population. The methodological framework is generalisable and can be applied to any condition for which PCOs and cost data are available.

To the best of our knowledge, this is the first proof-of-concept to estimate the value intended to be used as a patient-centred efficiency measure in VBHC. This paper puts forth two pivotal transformations of the value equation: the use of PACELY and the marginal perspective. These transformations are proposed to bring it into play as a decision-making tool in VBHC. Benchmarking based on PROs has been provided (5, 19) so far to monitor health outcomes to detect differences either across healthcare centres or patient archetypes based on their therapeutic pathways. This information might be useful to intermediate healthcare managers to identify best practices and improve the quality of healthcare providers, or to clinicians to implement shared decision-making as routine in clinical practice. The estimated figure may facilitate either holistic and comprehensive benchmarking across healthcare centres or disparate patient subgroups, combining health outcomes and costs in terms of value. This efficiency figure should provide valuable evidence to support reshaping the reimbursement systems and the implementation of value-based payment frameworks in VBHC (20) by the healthcare managers. Furthermore, the methods of this research might encourage researchers to advance VBHC in other medical conditions. In this context, a feasible standard set and a systematic collection of PCOs and EROs should be defined for each specific disease. Further research should address the estimation of VBHC thresholds and use the results presented in this paper to design value-based payment schemes.

## Data Availability

Raw data for dataset are not publicly available to preserve individuals’ privacy under the European General Data Protection Regulation. However, anonymized data might be shared upon author request.

## Group member of the VOICE study group

Members of the VOICE study group not listed among the co-authors are Susana Castelo Zas, Patricia Cobos Baena, Inés Gallego Camiña, Begoña Izaguirre Narbaiza, Gaizka Mallabiabarrena, Jon Cacicedo, Andere Frías, Alina Rigabert, William Balzi, Roberta Maltoni, Ilaria Massa, Isabel Álvarez López, Sara Arévalo Lobera, Mónica Esteban, Marta Fernández Calleja, Jenifer Gómez Mediavilla, Manuela Fernández, Manuel del Oro Hitar, María del Carmen Ortega Torres, María Consuelo Sanz Ferrandez, Luís Manso Sánchez, Pablo Serrano Balazote, Mario Campone, Sophie Le Lann, Piet Vercauter, Kurt Tournoy, Marina Borges, Ana Sofía Oliveira, Marta Soares, Iratxe Vázquez Lerma, Izaskun Artola Irazabal, Aitor Fernandez de Larrinoa Santamaría, Eduardo Vicario Elorduy, Valentina Danesi, Nicola Gentili, Andrea Roncadori, Fátima Hermoso Alarz, Karmele Imaz Iraola, Valerie Adam, Helene De Rijck, Ellen Everaert, Esmeralda Barreira, Pedro Medeiros and Emanuel Barros.
