# Supplementary material for: Bringing the value equation into play in value-based healthcare
Source: Front Public Health. 2026 Apr 13;14:1696854. doi: 10.3389/fpubh.2026.1696854 (PMC13112487; doi:10.3389/fpubh.2026.1696854)
Supplement: Supplementary file 1 [file Data_Sheet_1.docx]

**Table S1. Codebook of Economic-Related Outcomes^a^**

| **Resource use** | **Label** | **Value** |
| --- | --- | --- |
| visit_gine | Number of gynaecologist visit | 1, 2, ….N |
| visit_anest | Number of anesthesist visit |  |
| visit_onco | Number of oncologist visit |  |
| visit_surg | Number of surgeon visit |  |
| visit_radio | Number of radiotherapist visit |  |
| visit_rehab | Number of rehabilitator visit |  |
| visit_pyscho | Number of psychologist visit |  |
| visit_pharma | Number of pharmacist visit |  |
| visit_endo | Number of endocrinologist visit |  |
| bexam | Number of breast examinations |  |
| labtest_b | Number of laboratory basic profile tests |  |
| labtest_cea_ | Number of carcinoembryonic antigen (CEA) tests |  |
| labtest_ca | Number of carbohydrate antigen (CA) 15-3 tests |  |
| mamo_uni | Number of unilateral mamographies |  |
| mamo_bi | Number of bilateral mamographies |  |
| radio_th | Number of thorax radiographies |  |
| eco_abdo | Number of abdominal ecographies |  |
| eco_abdo_pel | Number of abdominal-pelvic ecographies |  |
| mri_breast | Number of breast magnetic resonances |  |
| mri_breast_con | Number of breast magnetic resonances with contrast |  |
| ct_th_abdo_pel | Number of thorax-abdomen-pelvis - Computerized tomographies |  |
| bone_densi | Number of bone densitometries |  |
| bone_scint | Number of bone scintigraphies |  |
| sln_tec | Number of sentinel lymph node using isotopic mapping (Tecnecium) |  |
| sln_blue | Number of sentinel lymph node using isotopic mapping (Blue dye) |  |
| sln_ferro | Number of sentinel lymph node using isotopic (Ferromagnetism) |  |
| biop_stero | Number of stereotactic biopsies |  |
| biop_core | Number of core needle biopsies |  |
| biop_core_ultra | Number of core needle ultrasound guided biopsies |  |
| biop_derma | Number of dermatologic punch key biopsies |  |
| biop_asp | Number of fine needle aspiration cytology (FNAC) biopsies |  |
| biop_test | Number of biopsy tests (pathological anatomy) |  |
| cito_test | Number of citology tests |  |
| electro | Number of electrocardiograms |  |
| ecocar | Number of ecocardiograms |  |
| oncotype | Number of oncotype tests |  |
| anest | Anesthesia (Minutes of operating room) |  |
| csurg | Number of conservative surgeries |  |
| csurg_min | Conservative surgery (Minutes of operating room) |  |
| mast | Number of mastectomy without reconstruction |  |
| mast_min | Mastectomy without reconstruction (Minutes of operating room) |  |
| mast_recon | Number of mastectomies with reconstruction |  |
| mast_recon_min | Mastectomy with reconstruction (Minutes of operating room) |  |
| prosthese | Number of breast prostheses |  |
| urpa | Postanesthetic Recovery Unit (Lenght of stay: minutes) |  |
| icu | Intensive care unit hospitalization (Lenght of stay: days) |  |
| hosp_conv | Conventional hospitalization (Lenght of stay: days) |  |
| hosp_home | Home hospitalization (Lenght of stay: days) |  |
| radio_session | Number of radiotherapy sessions |  |
| dhosp_session | Number of day hospital sessions |  |
| rehab_session | Number of rehabilitation sessions |  |
| CHEMO | "Yes" if patient has been prescribed with chemotherapy; "No" otherwise | No: 0; Yes: 1 |
| abemaciclib | "Yes" if patient has been prescribed with the active principe; "No" otherwise | No: 0; Yes: 1 |
| aterozolizumab | "Yes" if patient has been prescribed with the active principe; "No" otherwise |  |
| bevacizumab | "Yes" if patient has been prescribed with the active principe; "No" otherwise |  |
| capecitabine | "Yes" if patient has been prescribed with the active principe; "No" otherwise |  |
| carboplatin | "Yes" if patient has been prescribed with the active principe; "No" otherwise |  |
| cisplatin | "Yes" if patient has been prescribed with the active principe; "No" otherwise |  |
| cyclophosphamide | "Yes" if patient has been prescribed with the active principe; "No" otherwise |  |
| docetaxel | "Yes" if patient has been prescribed with the active principe; "No" otherwise |  |
| doxorubicin/adriamycin | "Yes" if patient has been prescribed with the active principe; "No" otherwise |  |
| doxorubicin (Pegylated Liposomal ) | "Yes" if patient has been prescribed with the active principe; "No" otherwise |  |
| doxorubicin (No pegylated Liposomal ) | "Yes" if patient has been prescribed with the active principe; "No" otherwise |  |
| epirubicin | "Yes" if patient has been prescribed with the active principe; "No" otherwise |  |
| fluorouracil (5-FU) | "Yes" if patient has been prescribed with the active principe; "No" otherwise |  |
| gencitabine | "Yes" if patient has been prescribed with the active principe; "No" otherwise |  |
| lapatinib | "Yes" if patient has been prescribed with the active principe; "No" otherwise |  |
| paclitaxel | "Yes" if patient has been prescribed with the active principe; "No" otherwise |  |
| palbociclib | "Yes" if patient has been prescribed with the active principe; "No" otherwise |  |
| pertuzumab | "Yes" if patient has been prescribed with the active principe; "No" otherwise |  |
| trastuzumab | "Yes" if patient has been prescribed with the active principe; "No" otherwise |  |
| trastuzumab Emtansine | "Yes" if patient has been prescribed with the active principe; "No" otherwise |  |
| vinorelbine | "Yes" if patient has been prescribed with the active principe; "No" otherwise |  |
| HORMO | "Yes" if patient has been prescribed with hormonotherapy; "No" otherwise | No: 0; Yes: 1 |
| anastrozole | "Yes" if patient has been prescribed with the active principe; "No" otherwise | No: 0; Yes: 1 |
| exemestane | "Yes" if patient has been prescribed with the active principe; "No" otherwise |  |
| goserelin | "Yes" if patient has been prescribed with the active principe; "No" otherwise |  |
| letrozole | "Yes" if patient has been prescribed with the active principe; "No" otherwise |  |
| leuprorelin | "Yes" if patient has been prescribed with the active principe; "No" otherwise |  |
| tamoxifen | "Yes" if patient has been prescribed with the active principe; "No" otherwise |  |
| SUPPORT | "Yes" if patient has been prescribed with support medication; "No" otherwise | No: 0; Yes: 1 |
| aprepitant | "Yes" if patient has been prescribed with the active principe; "No" otherwise | No: 0; Yes: 1 |
| denosumab | "Yes" if patient has been prescribed with the active principe; "No" otherwise |  |
| dexamethasone | "Yes" if patient has been prescribed with the active principe; "No" otherwise |  |
| epoetin alfa | "Yes" if patient has been prescribed with the active principe; "No" otherwise |  |
| filgastrim | "Yes" if patient has been prescribed with the active principe; "No" otherwise |  |
| netoclopramide | "Yes" if patient has been prescribed with the active principe; "No" otherwise |  |
| ondansetron | "Yes" if patient has been prescribed with the active principe; "No" otherwise |  |
| zolendronico | "Yes" if patient has been prescribed with the active principe; "No" otherwise |  |
| **Pharmacy costs** |  |  |
| c_chemo | Cost of the total chemotherapy drugs (including support medication) | Euros |
| c_hormo | Cost of the total hormonotherpay drugs |  |
| **Unit costs** |  |  |
| c_fvisit_gine | Cost of the first gynaecologist visit | Euros |
| c_fvisit_anest | Cost of the first anesthesist visit |  |
| c_fvisit_onco | Cost of the first oncologist visit |  |
| c_fvisit_surg | Cost of the first surgeon visit |  |
| c_fvisit_radio | Cost of the first radiotherapist visit |  |
| c_fvisit_rehab | Cost of the first rehabilitator visit |  |
| c_fvisit_pyscho | Cost of the first psychologist visit |  |
| c_fvisit_pharma | Cost of the first pharmacist visit |  |
| c_fvisit_endo | Cost of the first endocrinologist visit |  |
| c_svisit_gine | Cost of the succesive gynaecologist visit |  |
| c_svisit_anest | Cost of the succesive anesthesist visit |  |
| c_svisit_onco | Cost of the succesive oncologist visit |  |
| c_svisit_surg | Cost of the succesive surgeon visit |  |
| c_svisit_radio | Cost of the succesive radiotherapist visit |  |
| c_svisit_rehab | Cost of the succesive rehabilitator visit |  |
| c_svisit_pyscho | Cost of the succesive psychologist visit |  |
| c_svisit_pharma | Cost of the succesive pharmacist visit |  |
| c_svisit_endo | Cost of the succesive endocrinologist visit |  |
| c_bexam | Cost of the breast examination |  |
| c_labtest_b | Cost of the laboratory basic profile test |  |
| c_labtest_cea_ | Cost of the carcinoembryonic antigen (CEA) test |  |
| c_labtest_ca | Cost of the carbohydrate antigen (CA) 15-3 test |  |
| c_mamo_uni | Cost of the unilateral mamography |  |
| c_mamo_bi | Cost of the bilateral mamography |  |
| c_radio_th | Cost of the thorax radiography |  |
| c_eco_abdo | Cost of the abdominal ecography |  |
| c_eco_abdo_pel | Cost of the abdominal-pelvic ecography |  |
| c_mri_breast | Cost of the breast magnetic resonance |  |
| c_mri_breast_con | Cost of the breast magnetic resonance with contrast |  |
| c_ct_th_abdo_pel | Cost of the thorax-abdomen-pelvis - Computerized tomography |  |
| c_bone_densi | Cost of the bone densitometry |  |
| c_bone_scint | Cost of the bone scintigraphy |  |
| c_sln_tec | Cost of the sentinel lymph node using Isotopic mapping (Tecnecium) |  |
| c_sln_blue | Cost of the sentinel lymph node using Isotopic mapping (Blue dye) |  |
| c_sln_ferro | Cost of the sentinel lymph node using Isotopic mapping (Ferromagnetism) |  |
| c_biop_stero | Cost of the stereotactic biopsy |  |
| c_biop_core | Cost of the core needle biopsy |  |
| c_biop_core_ultra | Cost of the core needle ultrasound guided biopsy |  |
| c_biop_derma | Cost of the dermatologic punch key biopsy |  |
| c_biop_asp | Cost of the fine needle aspiration cytology (FNAC) biopsy |  |
| c_biop_test | Cost of the biopsy test (pathological anatomy) |  |
| c_cito_test | Cost of the citology test |  |
| c_electro | Cost of the electrocardiogram |  |
| c_ecocar | Cost of the ecocardiogram |  |
| c_oncotype | Cost of the oncotype test |  |
| c_anest_min | Cost of the minute of operating room |  |
| c_csurg_min | Cost of the minute of operating room |  |
| c_mast_min | Cost of the minute of operating room |  |
| c_mast_recon_min | Cost of the minute of operating room |  |
| c_prosthese | Cost of the breast prosthese |  |
| c_urpa | Cost of the minute at the Postanesthetic Recovery Unit |  |
| c_icu | Cost of the day at the Intensive care unit |  |
| c_hosp_conv | Cost of the day at the conventional hospital room |  |
| c_hosp_home | Cost of the day at the home hospitalization |  |
| c_radio_session | Cost of the radiotherapy session |  |
| c_dhosp_session | Cost of the day hospital session |  |
| c_rehab_session | Cost of the rehabilitation session |  |

^a^From García-Lorenzo et al.(5)

**Figure S1. Incremental Value Plane. Theoretical approach^a^**


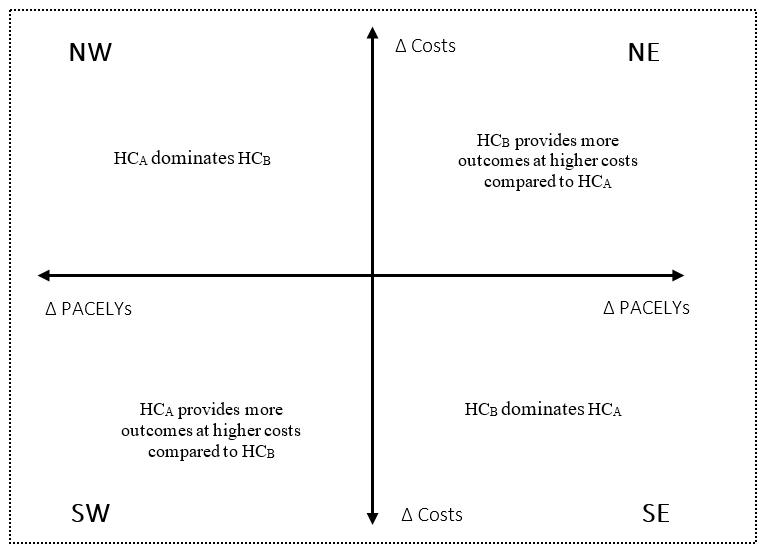


NW: Northwest; NE: Northeast; SW: Southwest; SE: Southeast; PACELY: Patient-Centred Outcome-Adjusted Life Years;

HC_A_: Healthcare centre A; HC_B_: Healthcare centre B

^a^ HC_B_ compared to HC_A_ being HC_A_ the reference

**Table S2. Descriptive analysis^a^**

|  | **Total**  **N=298**  **n (%)** | **HC_A_**  **N=52 (18.1%)**  **n (%)** | **HC_B_**  **N=246 (81.9%)**  **n (%)** | **P-value^b^** |
| --- | --- | --- | --- | --- |
| **PATIENT CHARACTERISTICS** | | | | |
| Age at diagnosis: (N=298) |  |  |  | 0.166 |
| ≤ 50 | 90 (30.2%) | 19 (36.5%) | 71 (28.9%) |  |
| 51-70 | 161 (54.0%) | 22 (42.3%) | 139 (56.5%) |  |
| > 70 | 47 (15.8%) | 11 (21.2%) | 36 (14.6%) |  |
| Educational Level: (N=274) |  |  |  | 0.148 |
| None | 17 (6.20%) | 0 (0.00%) | 17 (7.56%) |  |
| Primary | 79 (28.8%) | 12 (24.5%) | 67 (29.8%) |  |
| Secondary | 90 (32.8%) | 19 (38.8%) | 71 (31.6%) |  |
| Tertiary | 88 (32.1%) | 18 (36.7%) | 70 (31.1%) |  |
| Post-menopause status (N=294) | 192 (65.3%) | 30 (60.0%) | 162 (66.4%) | 0.483 |
| Comorbidity^c^ (N=296) | 110 (37.2%) | 13 (26.0%) | 97 (39.4%) | 0.103 |
| **TUMOUR CHARACTERISTICS** | | | | |
| Ductal carcinoma in situ (N=298) | 45 (15.1%) | 7 (13.5%) | 38 (15.4%) | 0.881 |
| Invasive ductal carcinoma (N=298) | 211 (70.8%) | 37 (71.2%) | 174 (70.7%) | 1 |
| Invasive lobular carcinoma (N=298) | 24 (8.05%) | 8 (15.4%) | 16 (6.50%) | 0.044 |
| Other carcinoma (N=298) | 18 (6.04%) | 0 (0.00%) | 18 (7.32%) | 0.044 |
| Positive estrogen receptor status (N=298) | 261 (87.6%) | 43 (82.7%) | 218 (88.6%) | 0.344 |
| Positive progesterone receptor status (N=297) | 237 (79.8%) | 35 (68.6%) | 202 (82.1%) | 0.046 |
| Positive HER2 receptor status: (N=297) | 26 (8.75%) | 5 (9.62%) | 21 (8.57%) | 0.951 |
| **TREATMENT CHARACTERISTICS** | | | | |
| Surgery: (N=297) |  |  |  | <0.001 |
| BCS | 227 (76.4%) | 31 (60.8%) | 196 (79.7%) |  |
| BCS with mammoplasty | 4 (1.35%) | 0 (0.00%) | 4 (1.63%) |  |
| Mastectomy without immediate reconstruction | 18 (6.06%) | 11 (21.6%) | 7 (2.85%) |  |
| Mastectomy with immediate reconstruction | 48 (16.2%) | 9 (17.6%) | 39 (15.9%) |  |
| Surgery to axilla: (N=297) |  |  |  |  |
| None | 27 (9.09%) | 5 (9.80%) | 22 (8.94%) |  |
| Sentinel lymph node biopsy | 220 (74.1%) | 38 (74.5%) | 182 (74.0%) | 0.977 |
| Axillary clearance | 50 (16.8%) | 8 (15.7%) | 42 (17.1%) |  |
| Radiotherapy (N=298) | 255 (85.6%) | 37 (71.2%) | 218 (88.6%) | 0.002 |
| Chemotherapy (N=298) | 92 (30.9%) | 18 (34.6%) | 74 (30.1%) | 0.633 |
| Hormonal therapy (N=298) | 258 (86.6%) | 46 (88.5%) | 212 (86.2%) | 0.83 |
| Targeted therapy (N=295) | 23 (7.80%) | 5 (9.80%) | 18 (7.38%) | 0.564 |
| **CROs** |  |  |  |  |
| Overall survival rate (N=298) | 298 (100% | 52 (100%) | 246 (100%) | 1 |
| Disease Free Survival (N=298) | 298 (100%) | 52 (100%) | 246 (100%) | 1 |
| Loco-regional recurrence (N=298) | 0 (0.0%) | 0 (0.0%) | 0 (0.0%) | . |
| Serious treatment-related complications (N=298) | 28 (9.40%) | 6 (11.5%) | 22 (8.94%) | 0.583 |
| **PROs** | **Mean (SD)** | **Mean (SD** | **Mean (SD** |  |
| Overall well-being (N=298) | 77.0 (17.2) | 73.1 (17.7) | 77.8 (17.0) | 0.08 |
| Physical functioning (N=298) | 87.9 (16.0) | 85.0 (15.8) | 88.5 (16.0) | 0.155 |
| Emotional functioning (N=298) | 85.9 (16.4) | 85.9 (14.8) | 85.9 (16.8) | 0.992 |
| Cognitive functioning (N=298) | 92.1 (15.8) | 88.5 (13.8) | 92.8 (16.1) | 0.048 |
| Social functioning (N=296) | 91.6 (17.8) | 82.1 (23.8) | 93.6 (15.6) | 0.001 |
| Ability to work (N=298) | 89.7 (20.2) | 83.3 (22.4) | 91.1 (19.4) | 0.024 |
| Finantial impact^d^ (N=298) | 5.26 (16.1) | 8.97 (17.6) | 4.47 (15.7) | 0.093 |
| Fatigue^d^ (N=298) | 20.1 (22.5) | 22.6 (24.4) | 19.6 (22.1) | 0.409 |
| Pain^d^ (N=298) | 17.8 (22.8) | 15.7 (24.3) | 18.3 (22.4) | 0.483 |
| Insomnia^d^ (N=296) | 27.7 (27.7) | 26.9 (26.4) | 27.9 (28.0) | 0.817 |
| Body image (N=295) | 89.0 (20.1) | 87.1 (21.0) | 89.4 (20.0) | 0.473 |
| Sexual functioning (N=255) | 22.2 (24.8) | 27.2 (26.8) | 21.1 (24.2) | 0.159 |
| Breast symptoms^d^ (N=296) | 11.5 (13.6) | 7.68 (11.9) | 12.2 (13.8) | 0.018 |
| Arm symptoms^d^ (N=298) | 9.28 (14.1) | 10.5 (13.3) | 9.03 (14.2) | 0.486 |
| Breast satisfaction(N=188) | 57.3 (9.50) | 55.5 (8.52) | 57.9 (9.75) | 0.125 |
| Peripheral symptoms^d^ (N=296) | 16.2 (24.7) | 19.9 (24.9) | 15.4 (24.6) | 0.247 |
| Vaginal symptoms^e^ (N=294) | 18.3 (2.15) | 19.2 (1.75) | 18.1 (2.18) | <0.001 |
| Arthralgia^f^ (N=298) | 3.02 (1.18) | 3.19 (0.93) | 2.98 (1.23) | 0.162 |
| Vasomotor symptoms^d^ (N=296) | 31.2 (32.9) | 32.1 (33.0) | 31.0 (33.0) | 0.837 |
| **EROs** |  |  |  |  |
| Specialist visits | 23.65 (10.89) | 26.51 (13.23) | 23.04 (10.26) | 0.033** |
| Diagnostic tests | 28.27 (19.98) | 33.92 (16.56) | 27.07 (20.46) | 0.024** |
| Hospital length of stay | 8.40 (9.20) | 6.45 (6.04) | 8.95 (9.87) | 0.182 |
| Day hospital sessions | 10.22 (7.60) | 20 (6.91) | 8.42 (6.25) | 0.000*** |
| Radiotherapy sessions | 36.67 (7.07) | 45.96 (13.08) | 35.21 (3.98) | 0.000*** |
| Diagnostic costs | 3,706 (1941) | 2,859 (1140) | 3,885 (2028) | 0.000*** |
| Inpatient costs^g^ | 5,055 (4555) | 3,742 (3510) | 5,606 (4663) | 0.007*** |
| Pharmacologic Treatment costs^h^ | 9,293 (9357) | 1,984 (4,498) | 2,953 (7662) | 0.000*** |

PRO: Patient-Reported Outcome; CRO: Clinical-Related Outcome; ERO: Economic-Related Outcome; BCS: Breast Cancer Surgery; HC_A_: Healthcare centre A; HC_B_: Healthcare centre B

^a^Percentages calculated excluding missing data.

^b^P-value corresponding to the chi-square test.

^c^Comorbidity has been defined as a binary variable equal to 1 if patient presents a comorbidity.

^d^High score represents high level of symptomatology

^e^Scale ranges between 0 and 20

^f^Scale ranges between 0 and 4

^g^ Includes surgery and hospital stay costs.

^h^ Includes chemotherapy and hormonotherapy costs.

***p-value < 0.01; **p-value<0.00
